# Educ’ Alcool’s misinformation: more mixed messages about alcohol harms

**DOI:** 10.1093/eurpub/ckab198

**Published:** 2022-02-01

**Authors:** Mark P Petticrew, May C I van Schalkwyk, Nason J Maani, Lewis K Peake

**Affiliations:** Faculty of Public Health and Policy, London School of Hygiene and Tropical Medicine, London, UK

Our study, which focussed on the cardiovascular disease (CVD) risks posed by alcohol, builds on previous evidence in analyzing how efforts to address public health threats, including alcohol harms, may be undermined by commercial actors.^1^ Previous research across many harmful products documents how corporate social responsibility activities form a critical arm of efforts in fomenting doubt about product harms.^2^^,^^3^

While we welcome constructive critique of our work, Mr Sacy does not appear to have understood how we conducted our research. The Methods section explicitly states that webpage content constituted the dataset, and that websites were accessed during June 2019. The table which he presents forms part of a PDF report, not a public facing webpage where people can readily access key facts on alcohol and CVD.

Mr Sacy also claims that his information is unbiased, and similar to non-industry-funded information. Contrary to this statement, we have analyzed Educ’ Alcool’s materials as part of multiple larger studies of alcohol industry misinformation. It is clear that industry-funded organizations including Educ’ Alcool selectively misinform the public about pregnancy harms, cancer and now CVD.^4–6^ This is consistent with evidence on the nature, function and effects of alcohol industry corporate social responsibility efforts more generally.^7^

The Educ’ Alcool report, which Mr Sacy cites in his response, is itself very problematic. It contains framings consistent with many of the misinformation techniques which we have previously documented. These include the strong selective positive framing of alcohol consumption, and overclaiming of the benefits of alcohol. For example, discussion of ‘Harmful effects’ features on p9, after multiple sections detailing ‘Helpful Effects’ and ‘Protective Effects’. This sort of ‘nudging’ of readers away from clear evidence on harms, while foregrounding benefits, is a common alcohol industry misinformation tactic.^8^

His table is also concerning, consisting of a cherry-picked, unreferenced selection of five studies; it is unclear how this is supposed to represent the wider evidence base. It is also unclear what purpose it is intended to serve, but it certainly does not represent any meaningful or unbiased representation of the evidence.

The tone and content of Mr Sacy’s comments may surprise readers, but are consistent with research by Bartlett and McCambridge, who recently analyzed how alcohol industry and related actors aggressively respond to criticism; their response is characterized by ‘making narrow claims about accuracy while ignoring substantial engagement with the issues of framing, context, and impacts on readers … The SAO [AI-funded Social Aspects Organisations] interventions are thus highly defensive, designed to protect the reputations of the organizations. The replies, printed in peer-reviewed journals, thus operate as public relations exercises given legitimacy by being located within the scientific literature …’.^9^

Edu’alcool’s materials and Mr Sacy’s response are entirely consistent with the growing evidence on alcohol misinformation and what has been called its ‘strategic ambiguity’.^10^ As we concluded previously, independent bodies (such as government health departments) should not use or signpost to material from SAPRO’s, given that it has the characteristics of other unhealthy commodity industry-funded misinformation, and significantly misrepresents the evidence. 

## Funding

N.J.M. is supported by a Harkness Fellowship in Healthcare Policy and Practice from the Commonwealth Fund. M.C.I.v.S. acknowledges funding from the National Institute for Health Research Doctoral Fellowship (NIHR300156). M.P.P. and N.J.M. are investigators in the SPECTRUM Consortium, which is funded by the UK Prevention Research Partnership, a consortium of UK funders [UK Research and Innovation Research Councils: Medical Research Council, Engineering and Physical Sciences Research Council, Economic and Social Research Council, and Natural Environment Research Council; charities: British Heart Foundation, Cancer Research UK, Wellcome, and The Health Foundation; government: Scottish Government Chief Scientist Office, Health and Care Research Wales, National Institute of Health Research (NIHR), and Public Health Agency (NI)]. Grant Reference: UKPRP_CO1_103. 

*Conflicts of interest*: None declared. 
